# Complete Genome Sequence of the Acetic Acid Bacterium Acetobacter aceti NBRC 14818

**DOI:** 10.1128/MRA.01039-20

**Published:** 2020-10-22

**Authors:** Hiroyuki Arai, Masafumi Kameya, Masaharu Ishii

**Affiliations:** a Department of Biotechnology, Graduate School of Agricultural and Life Sciences, The University of Tokyo, Bunkyo-ku, Tokyo, Japan; b Collaborative Research Institute for Innovative Microbiology, The University of Tokyo, Bunkyo-ku, Tokyo, Japan; University of Southern California

## Abstract

We report here the complete genome sequence of the acetic acid bacterium Acetobacter aceti type strain NBRC 14818. The genome comprises a chromosome of 3,596,270 bp with 57.1% GC content and four plasmids/phages of 63,279 bp, 25,755 bp, 4,858 bp, and 2,964 bp, with an average GC content of 57.0%.

## ANNOUNCEMENT

The acetic acid bacterium Acetobacter aceti, the type species of the genus *Acetobacter*, has been utilized for vinegar production and causes the spoilage of alcoholic beverages due to its unique ability to incompletely oxidize ethanol to acetate (1, 2). NBRC 14818 (https://www.nite.go.jp/nbrc/catalogue/NBRCCatalogueDetailServlet?ID=NBRC&CAT=00014818) (ATCC 15973, DSM 3508, JCM 7641), the type strain of *A. aceti*, was isolated from an alcoholic beverage that had turned to vinegar. Transcriptome profiles of *A. aceti* under various growth conditions have been investigated based on our draft genome sequence of NBRC 14818, determined with the Illumina Genome Analyzer II (2 – 5). However, its complete genome sequence had not previously been reported. We report here the complete genome sequence, consisting of a single chromosome and four plasmids/phages.

Whole-genome DNA isolated from cells grown in ethanol medium (3) according to the method of Marmur (6) in the previous study for the draft genome analysis (3) was used for sequencing with the Pacific Biosciences (PacBio) RS II platform (7). A single-molecule real-time (SMRT) cell library was constructed with 8 μg of DNA using the PacBio 20-kb protocol. Default settings were used for all software unless otherwise noted. PacBio sequencing and read quality control were performed with SMRT Portal version 2.3.0 (PacBio). The library yielded 135,575 subreads (adaptor-trimmed reads) with a mean length and *N*_50_ value of 9,197 bp and 12,944 bp, respectively. The genome coverage was 331-fold based on the complete genome sequence. The subreads were *de novo* assembled using Canu version 1.0.6 (8) with the options “genomeSize=3.7m -pacbio-raw,” producing 6 contigs with a total length of 3.769 Mb. The contigs were error corrected with the Illumina reads of the previous study (3) by using Genetyx software version 13 (Genetyx Co.). The corrected sequences were circularized by removing the overlapping ends manually into a chromosome of 3,596,270 bp with a GC content of 57.1% and two possible plasmids or phages of 63,279 bp and 25,755 bp, designated pAACEN1 and pAACEN2, with GC contents of 57.1% and 56.7%, respectively. Of the 1,488 Illumina contigs (3), which were assembled using Edena version 2.1.1 software (9), 1,478 were mapped in the assembled sequences using BLASTN version 2.7.1+ (10). The remaining 10 contigs were connected by brute-force PCR amplification with primers designed near the ends of the contigs, and the PCR fragments were sequenced with the ABI 3730xl DNA analyzer (Applied Biosystems). As a result, the contigs were manually assembled into two circular plasmids of 4,858 bp and 2,964 bp, designated pAACEN3 and pAACEN4, with GC contents of 58.0% and 56.4%, respectively. The genes were annotated using the DDBJ Fast Annotation and Submission Tool (DFAST) (https://dfast.nig.ac.jp/) with ARAGORN to predict the tRNA genes. The chromosome and plasmid/phage sequences were rotated to start at the *dnaA* gene and the predicted genes involved in replication, respectively. The chromosome was found to contain 3,293 predicted protein-coding sequences (CDSs), 3 sets of rRNA genes, and 51 tRNA genes. pAACEN1, pAACEN2, pAACEN3, and pAACEN4 were found to contain 69, 39, 8, and 5 CDSs, respectively.

### Data availability.

The complete genome sequence of *A. aceti* NBRC 14818 has been deposited at DDBJ under accession numbers AP023410 (chromosome), AP023411 (pAACEN1), AP023412 (pAACEN2), AP023413 (pAACEN3), and AP023414 (pAACEN4). The raw sequence data have been deposited at DDBJ Sequence Read Archive under the accession number DRA010690. The sequences of the primers used for PCR and Sanger sequencing of the plasmids are available on Figshare at https://doi.org/10.6084/m9.figshare.13055402.
